# Nontoxic concentration of ochratoxin A decreases the dosage of cyclosporine A to induce chronic nephropathy model via autophagy mediated by toll-like receptor 4

**DOI:** 10.1038/s41419-020-2353-z

**Published:** 2020-02-27

**Authors:** Lili Hou, Guannan Le, Ziman Lin, Gang Qian, Fang Gan, Cong Gu, Shuai Jiang, Jiaxin Mu, Lei Ge, Kehe Huang

**Affiliations:** 1 College of Veterinary Medicine, Nanjing Agricultural University, Nanjing, 210095 Jiangsu Province China; 2 Institute of Nutritional and Metabolic Disorders in Domestic Animals and Fowls, Nanjing Agricultural University, Nanjing, 210095 Jiangsu Province China; 3 MOE Joint International Research Laboratory of Animal Health and Food Safety, College of Veterinary Medicine, Nanjing Agricultural University, Nanjing, 210095 Jiangsu Province China

**Keywords:** Diseases, Chronic kidney disease

## Abstract

Cyclosporine A (CsA) extracted from the products of fungal fermentation is used to develop a chronic nephropathy model. However, it has numerous side effects. Ochratoxin A (OTA) is a mycotoxin that induces renal injury. We developed a chronic nephropathy model to lessen the side effects of CsA by administration of nontoxic dosage of OTA, and investigated the underlying mechanism. C57BL/10 wild-type mice, toll-like receptor 4 (TLR4)^−/−^ mice, and HK-2 cells were used in this study. The nontoxic dosage (0.25 mg/kg, qod) of OTA could significantly decrease the dosage of CsA from 30 to 20 mg/kg per day, and combination of them induced chronic nephropathy model and alleviated the side effects of onefold CsA in vivo, including cardiotoxicity, hepatotoxicity, and immunosuppression. The nontoxic concentration (0.5 μg/ml) of OTA could significantly decrease the concentration of CsA from 10 to 6 μg/ml that induced cytotoxicity, oxidative stress, and nephrotoxicity *i*n vitro. Nontoxic concentration of OTA and low dosage of CsA activated TLR4 and autophagy. These toxic effects induced by OTA and CsA could be reversed by knockdown of TLR4 and autophagy inhibitor 3-methyladenine in vitro. Furthermore, the renal injury and autophagy induced by OTA and CsA could be attenuated in TLR4^−/−^ mice. It suggested that a chronic nephropathy model had been successfully developed by administration of nontoxic concentration of OTA and low dosage of CsA via TLR4-mediated autophagy. The side effects of current model were significantly lesser than those of the previous model induced by onefold CsA. It provided a new tool for exploring the pathogenesis and treatment of chronic kidney disease.

## Introduction

Cyclosporine A (CsA) extracted from the products of fungal fermentation is commonly used in organ transplant patients, since the early 1980’s. It mainly acts on the antirejection response of transplantation^1^. In this respect, CsA raises survival and quality of life in transplant patients. Any deviation from critical dose limit of CsA can result in severe toxicities or renal failure, although cyclosporine-microemulsion (CsA-ME) has been shown to be effective and safe^2^. CsA is often used to develop a chronic nephropathy model because of its nephrotoxicity^3–6^. Chronic CsA renal toxicity is characterized by pathological changes of tubular vacuolization and striped cortical interstitial fibrosis^7^. Chronic CsA nephropathy model facilitates a lot of studies and supports researchers to find out different ways of preventing CsA-induced renal injury and the underlying mechanism. However, long-term and massive intake of CsA has numerous complications. It has many restrictive side effects in the clinical application of CsA, such as hepatotoxicity, cardiotoxicity, neurotoxicity, and hypertension^8–10^. Based on these studies, we speculated whether the adverse effects of CsA on other tissues and organs could be mitigated by reducing the amount of CsA.

Ochratoxin is a mycotoxin that acquires worldwide attention after aflatoxin. It is an important kind of mycotoxins and produced by both *Aspergillus* and *Penicillium*^11,12^. Among this group, ochratoxin A (OTA) is a nephrotoxic member, which is main causative agent of human Balkan endemic nephropathy^13^. It was reported that OTA induced nephrotoxicity through MAPK signaling pathways and JAK2/STAT3 signaling pathway in PK15 cells^14,15^. More than that, a research showed that a higher survival rate and better biochemical parameters of the blood serum were observed in the group of piglets exposed to low dose of OTA than that in high dose of OTA^16^. Consequently, we hypothesized that nontoxic dose of OTA could probably decrease the dose of CsA to induce chronic nephropathy model as well.

It has been reported that autophagy, oxidative stress, and some signal pathways participated in nephrotoxicity induced by CsA^17^. The expression of transforming growth factor-beta1 (TGF-β1), excessive apoptosis, autophagy, and production of inflammatory factors could be upregulated in chronic CsA nephropathy^18^. OTA-induced nephropathy was related to them as well^19–21^. Among them, inflammation plays a vital role in the progression of renal diseases, in which toll-like receptor 4 (TLR4) represents a major component in innate immunity. It’s previously reported that as an important mechanism resulting in CsA-induced pro-inflammatory factors synthesis, TLR4 was activated in CsA nephropathy^22^. On the other hand, autophagy associated with renal injury was activated after the administration of CsA^17^. It has been reported that autophagy mediated by TLR4 made contribution to microglial activation and inflammatory injury in mice^23^. However, the underlying mechanisms about the nephrotoxicity induced by OTA and CsA in combination have not yet been illuminated.

The objectives are to eatablish a chronic nephropathy model with lesser side effects by nontoxic concentration of OTA and low dosage of CsA in combination than those of the previous model by alone CsA and explore their underlying mechanisms.

## Materials and methods

### Experimental animals and treatments

Male mice (6–8 weeks old, *n* = 110) were purchased from the Experimental Animal Center of Yangzhou University (Yangzhou, China). TLR4 knockout male mice (*n* = 30; C57BL/10ScNJNju, TLR4^−/−^), 6–8 weeks old were bought from GemPharmatech Co, Ltd (Nanjing, China). Due to the TLR4 lps-del mutation, functional TLR4 or TLR4 mRNA do not be expressed in TLR4^−/−^ mice. Before the experiment, 140 mice were kept in animal cages for 1 week under a constant light/dark cycle (12 h/12 h), temperature (23 ± 1 °C), and humidity (60 ± 10%), with free access to food and tap water. All procedures followed the Guide for the Care and Use of Laboratory Animals published by the National Institutes of Health (Bethesda, MD, USA) and approved by the Committee for the Care and Use of Experimental Animals at the Agriculture University of Nanjing (certification no.: SYXK (Su)2011–0036).

Firstly, mice were assigned into six groups randomly (control (group A), 0.25 mg/kg per day OTA (group B), 0.25 mg/kg per day OTA combined with 10 mg/kg per day CsA (group C), 20 mg/kg per day CsA (group D), 0.25 mg/kg per day OTA combined with 20 mg/kg per day CsA (group E), and 30 mg/kg per day CsA (group F)). Secondly, mice were divided into four groups (control, TLR4^−/−^, OTA (0.25 mg/kg per day) + CsA (20 mg/kg per day), and OTA + CsA + TLR4^−/−^). Each group had two replicates with six mice per replicates. OTA and CsA were intraperitoneally injected every other day and daily for 28 d, respectively.

### Detections of serum biochemistry and urine indexes

After treatment for 28 d, serum was collected. Blood urea nitrogen (BUN), serum creatinine (Scr), creatine kinase (CK), and lactate dehydrogenase (LDH), aspartate aminotransferase (AST), and alanine aminotransferase (ALT) in serum were detected using standard kits purchased from Jiancheng (Nanjing, China) according to the manufacturer’s illustrations. Urine gravity (UG) and urine protein (UP) were measured using hand-held refractometer (SUR-NE, Japan).

### Histopathological and immunohistochemical analysis

Tissue samples were fixed in 10% neutral buffered formalin, sectioned at a thickness 4 μm and processed for paraffin embedding. Hematoxylin and eosin (H&E) staining, Masson staining, and immunohistochemical analysis were used according to standard procedures^24^.

### Cell culture

Human renal proximal tubule epithelial (HK-2) cells were purchased from Gefan Biotechnology (Shanghai, China) and cultured in RPMI-1640 medium (Gibco, USA). Heat-inactivated 10% fetal bovine serum (FBS, Gibco, USA), and 1% antibiotics of penicillin and streptomycin were added into medium. Mycoplasma contamination is negative.

### MTT assay

Cells were seeded at a density of 4 × 10^3^ cells/well in the 96-well plates and exposed to CsA or OTA alone and in combination for 48 h. While 15 μL 3-(4, 5-dimethyl-2-thiazolyl)-2, 5-diphenyl-2-H-tetrazolium bromide,thiazolyl blue tetrazolium bromide (MTT) (5 mg/mL) was added into the culture medium at 37 °C for 3–4 h before the end of the treatment. Then the supernatants were abandoned and 150 μL dimethyl sulfoxide were added to dissolve the crystal. The absorbance was detected at 490 nm using a Microplate Reader (Thermo Fisher, USA). The optical density value of each group were obtained and expressed as percentages of control group. Six replications were performed.

### LDH activity

To detect LDH activity in vitro, cells were seeded at a density of 4 × 10^3^ cells/well in the 96-well plates with corresponding treatment. After treatment, the culture medium was collected and centrifuged. The supernatant was collected and stored it at −20 °C until analysis^25^. LDH assay kit bought from Jiancheng (Nanjing, China) was used to assess the LDH activity. The test was performed five replicates.

### Cell proliferation ability

Cells were incubated with corresponding treatment. After corresponding treatment, cells were cultured with EdU markers (10 μM) for 2 h and fixed by 4% paraformaldehyde for 15 min. Then cells were washed and penetrated. BeyoClick™ EdU cell proliferation test kit purchased from Beyotime Biotechnology (Haimen, China) was used to evaluated cell proliferation capacity. The results were scanned with an ordinary optical microscope (Nikon Instruments, Inc).

### Cell apoptosis assay

To investigate morphology of nuclear, 20-mm round coverslips (WHB, China) were used to culture cells in 12-well plates. After treatments, the cells were stained with Hoechst33258 (1 mg/mL) for 10 min. Finally, the slides were washed three times with phosphate-buffered saline (PBS) and scanned with a fluorescence microscope^25^.

### Determinations of intracellular ROS and glutathione

Cells were seeded on 20 mm round coverslips with corresponding treatment. After removing the cell culture medium, cells were stained with the protocol of Reactive Oxygen Species (ROS) Assay Kit (Beyotime, Shanghai, China). Cells were timely scanned by confocal microscopy. Intracellular ROS were labeled by green fluorescent light observed in the microscope. On the other hand, cells were collected and glutathione (GSH) was tested with the commercial reduced GSH assay kit (Jiancheng, Nanjing, China). GSH can react with disulfide dinitrobenzoic acid to produce a yellow compound, which can be used for colorimetric quantitative determination of GSH at 405 nm.

### Immunofluorescence assay by laser scanning confocal microscope

Confocal fluorescence microscopy was used for analyzing expressions of Vimentin and α-smooth muscle actin (α-SMA). The cells were washed with ice PBS and incubated in specific primary antibodies (anti-vimentin (bs-23063R) and anti-α-SMA (bs-10196R), Bioss Inc. Beijing, China). Then cells were washed again and incubated in fluorescein isothiocyanate-labeled anti-rabbit immunoglobulin G antibody (A0562, Beyotime, China) and 4′,6-diamidino-2-phenylindole (C1002, Beyotime, China). Vimentin and α-SMA that were fluorescent labeled could be visualized by the confocal microscope.

### Real-time PCR analysis

After corresponding treatment, RNA was extracted using trizol reagent. RNA samples, after quality (1.8 ≤ A260/A280 ≤ 2.0) and concentration evaluation using a NanoDrop 2000 spectrophotometer, were stored at −80 °C until used in the experiments. Single-stranded complementary DNA was reverse transcribed. The relative mRNA levels were detected by the Δ cycle threshold method. And β-actin served as the housekeeping gene. The primers (Table 1) were designed and synthesized by Sangon Biotech (Shanghai, China).

**Table 1:** List of genes’ forward and reverse primer sequences.

Target genes	Forward (5′–3′)	Reverse (5′–3′)
Mouse		
β-actin	AAATCGTGCGTGACATCAAA	ATGCCACAGGATTCCATACC
α-SMA	CTTCGTGACTACTGCCGAGC	AGGTGGTTTCGTGGATGCC
IL-10	GGTTGCCAAGCCTTATCGGA	GAGAAATCGATGACAGCGCC
LC3	GATGTCCGACTTATTCGAGAGC	TTGAGCTGTAAGCGCCTTCTA
TGF-β1	AGCTGCGCTTGCAGAGATTA	AGCCCTGTATTCCGTCTCCT
TLR4	GATAGCGAGCCACGCATTCA	TTAGGAACCACCTCCACGCAG
TNF-α	AGGCACTCCCCCAAAAGATG	CCACTTGGTGGTTTGTGAGTG
Vimentin	AGCAGTATGAAAGCGTGGCT	CTCCAGGGACTCGTTAGTGC
Human		
β-actin	GGTGGTCTCCTCTGACTTCAACA	GTTGCTGTAGCCAAATTCGTTGT
α-SMA	CCCTTGAGAAGAGTTACGAGTTG	ATGATGCTGTTGTAGGTGGTTTC
CTGF	GTGTGCACCGCCAAAGATGG	CCAACCACGGTTTGGTCCTT
E-cadherin	TGAGTGTCCCCCGGTATCTT	GAATCATAAGGCGGGGCTGT
Fibronectin	CTGGCCAGTCCTACAACCAG	CGGGAATCTTCTCTGTCAGCC
LC3	GTCCAACAACAGCACCATGC	TCTCCAAACAGCGTCTGGCT
TGF-β1	TACCTGAACCCGTGTTGCTC	CCGGTAGTGAACCCGTTGAT
TLR4	TATCCAGAGCCGTTGGTGTATCT	AATGAAGATGATGCCAGAGCG
Vimentin	AACTTAGGGGCGCTCTTGTC	CCTGCTGTCCCGCCG

### Western blotting analysis

The total protein was extracted and the concentration was measured by bicinchonininc acid (BCA) protein assay kits (Beyotime, China). Forty micrograms of protein were denatured and heated at 95 °C for 5 min. The denatured protein was transferred to polyvinylidene difluoride (PVDF) membranes after sodium dodecyl sulfate–polyacrylamide gel electrophoresis assay^25^. The membranes were incubated with specific primary antibodies, including anti-α-SMA, anti-Vimentin (bs-10196R and bs-23063R, Bioss, Beijing, China), anti-AKT, anti-p-AKT, anti-TGF-β1 (A11016, AP0140, A15103, ABclonal, Wuhan, China), anti-mTOR, anti-p-mTOR (ab2732 and ab109268, Abcam, Cambridge, UK), anti-LC3 (L8918, Sigma, St.Louis, MO, US), anti-ATG5, anti-TLR4 (sc-133158 and sc-293072, Santa, Texas, US), and anti-β-actin (4970, CST, MA, United States) at 4 °C overnight. The PVDF membranes were then incubated with horseradish peroxidase (HRP)-labeled anti-rabbit secondary antibody (A0208, Beyotime, China). Blots were obtained and expressed in percentage respect to the control group.

### Transfection of small interfering RNA

The TLR4-specific small interfering RNA (siRNA) sequence was 5′-UUCUAGUUGUUCUAAGCCCTT-3′. The sequence of control siRNA was 5′-ACGUGACACGUUCGGAGAATT-3′. These were synthesized by Invitrogen. Through using X-tremeGENE transfection reagent (Roche, Switzerland), TLR4-specific siRNA was transiently transfected into cells. In short, cells were cultured at 37 °C for 30–50% confluence. TLR4-specific siRNA was introduced according to the illustration. Transfection reagent and siRNA (5:1) were mixed and incubated with cells for 6 h. Finally, cells were washed with 1640 medium and switched to culture medium with 4% FBS for corresponding treatments^21^.

### Statistical analysis

Results were statistically analyzed by one-way analysis of variance, followed by Duncan's multiple range tests to analyze. All statistical analyses were performed with SPSS 18.0. Every experiment was performed at least three replicates, and the values were presented as mean ± standard deviation (SD). *P* *<* 0.05 was considered statistically significant and *P* *<* 0.01 was considered markedly significant (95% confidence interval). The variance is similar between the groups that are being statistically compared.

## Results

### Nontoxic dosage of OTA decreased the dosage of CsA to induce renal injury and alleviated the side effects of CsA in vivo

**Fig. 1:**
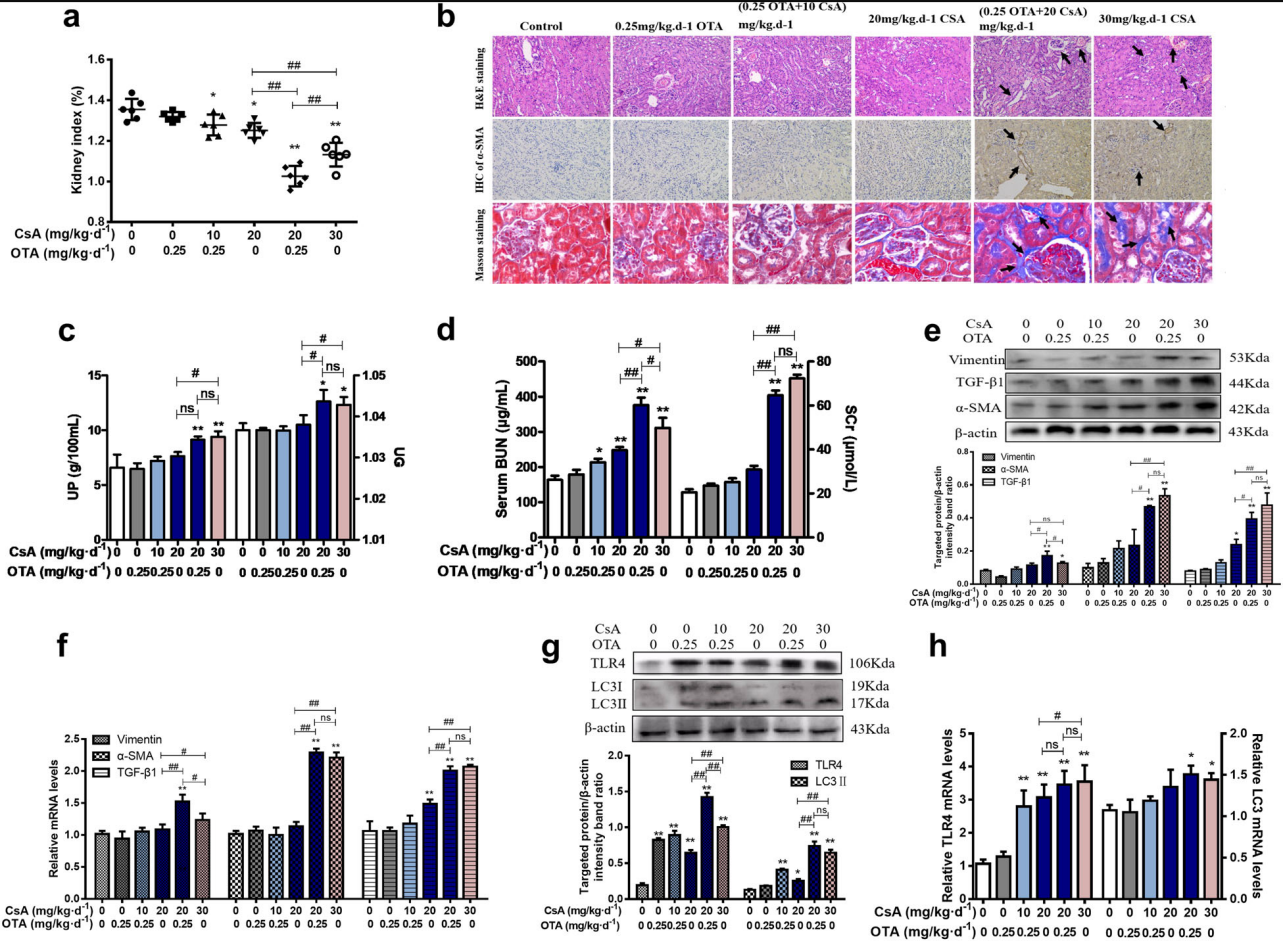
Nontoxic OTA decreased the dosage of CsA to induce renal injury. Mice were divided into six groups, treated with OTA or/and CsA for 28 days. Kidney index **a** were calculated as the ratio of kidney weight and body weight. Pathological changes **b** of H&E (100×) and Masson (400×) staining were observed in kidneys. UG and UP **c** were measured by hand-held refractometer. Serum BUN and Scr **d** were detected by corresponding kits. Renal injury related proteins **e** and relative mRNA levels **f** were tested by western blotting and real-time PCR. Expressions of TLR4 and LC3 were determined **g**, **h**. All results were expressed as means ± SD (*n* = 6, *N* = 3). Significance compared with the control group, *P < 0.05, **P < 0.01, ns, no significance; significance compared with the group of 30 mg/kg per day CsA, ^#^*P* *<* 0.05, ^##^*P* < 0.01, ns, no significance.

At the beginning of the experiment, appearance of the mice was normal. The pad was clean and free of odor. After treatment for 1 week, the pads of two combined groups and group of 30 mg/kg per day CsA were found to be wetter than that of the control group. Following treatment for 28 d, kidney index was apparently decreased and kidney injury was increased in the group of 0.5 mg/kg per day OTA as compared with the control group, and the group of 0.25 mg/kg per day OTA had no significant change (Supplementary Fig. 1). Therefore, the dosage (0.25 mg/kg per day) of OTA in group B was selected. As shown in Fig. 1a, kidney index of groups C, E, and F were significantly lower than that of control group (*P* < 0.05). H&E staining in groups E and F showed that partial renal tubular structural changes and a small number of inflammatory cells in the tubule interstitium as compared to control group (Fig. 1b). The positive area of Masson (blue) staining, which stained the renal fibrosis-related protein collagen I revealed significant increases in the groups E and F, whereas other three groups hadn’t the renal changes (Fig. 1b). Besides, UG and UP in Fig. 1c were found out significant increases in groups E and F (*P* < 0.05), but there was no significant difference between these two groups (*P* > 0.05). Additionally, we tested levels of serum BUN and Scr. The results showed that serum BUN level was significantly higher than group F, while Scr level had non-significant changes in group E as compared to group F (Fig. 1d). Renal fibrosis-related proteins and genes (TGF-β1, Vimentin, and α-SMA) were measured via immunohistochemical staining of α-SMA, real-time PCR, and western blotting. As shown in Fig. 1b, e, f, expressions of TGF-β1, α-SMA, and Vimentin were evidently ascended in group E (*P* < 0.05). In Fig. 1g, h, we found that expressions of TLR4 and LC3 were upregulated with the involvement of OTA and CsA in vivo. It indicated that group E could successfully induce renal damage, as well as group F that was a positive model.

**Fig. 2:**
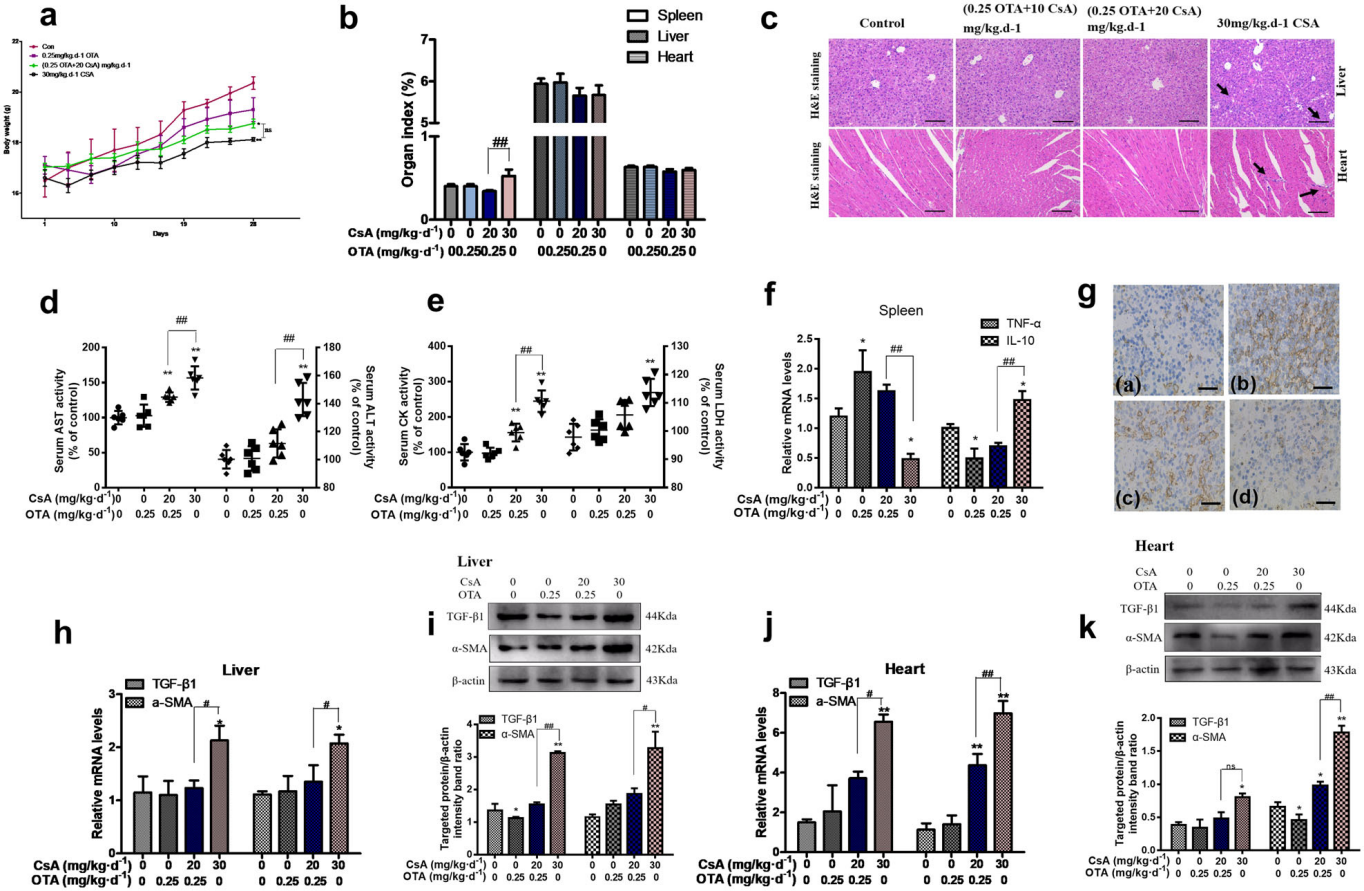
Nontoxic OTA and low dosage of CsA in combination alleviated the side effects of CsA in vivo. Mice were divided into six groups, treated with OTA or/and CsA for 28 days. Body weight was recorded **a**. Spleen, liver, and heart **b** indexes were calculated as the ratio of liver or cardiac weights and body weight. Pathological changes **c** of H&E staining (100×) were observed in livers and hearts. Specific cardiac and liver serum indexes were measured **d**, **e**. Expressions of inflammatory factors of spleen were detected by real-time PCR **f** and changes of immunohistochemical staining of CD3 were measured **g**. Liver and cardiac fibrosis-related proteins **i**, **k** and relative mRNA levels **h**, **j** were tested by western blotting and real-time PCR. All results were expressed as means ± SD (*n* = 6, *N* = 3). Significance compared with the control group, *P < 0.05; significance compared with the group of 30 mg/kg per day CsA, ^*#*^*P* < 0.05.

To further verify alleviation of the side effects, body weight, spleen, liver, and heart index were calculated as shown in Fig. 2a, b. However, no significant change was observed between groups E and F (*P* > 0.05). But more inflammatory cells and disorganization of the myofibrils were observed in heart (Fig. 2c). The wider hepatic intercellular space, cell disruption, and infiltration of large amount of inflammation cells were found in liver of group F. Obvious increases in serum AST and ALT, which represents liver damage, CK and LDH, which represents myocardial damage were observed in the group F, but their levels were decreased in group E (Fig. 2d, e). Expression of pro-inflammatory factor TNF-α in spleen was inhibited and expression of anti-inflammatory factor IL-10 in spleen was promoted in the group F (*P* < 0.05). But it was reversed in the group E (Fig. 2f). The blots of immunohistochemical staining of CD3 in the group F were less than that of group E (Fig. 2g). Particularly, clear rising expressions of TGF-β1 and α-SMA (*P* < 0.05) have been confirmed by real-time PCR (Fig. 2h, j) and western blotting (Fig. 2i, k) in group F compared with control group in both liver and heart. On the other hand, a mouse in group F was moving always in circle and that was a clear neurotoxicity indication (Video 1). It indicated that the dosage of CsA was reduced with involvement of nontoxic OTA, and the side effects of high dosage of CsA were significantly lessened as well.

### Nontoxic concentration of OTA decreased the concentration of CsA to induce cytotoxicity and oxidative stress in vitro

**Fig. 3:**
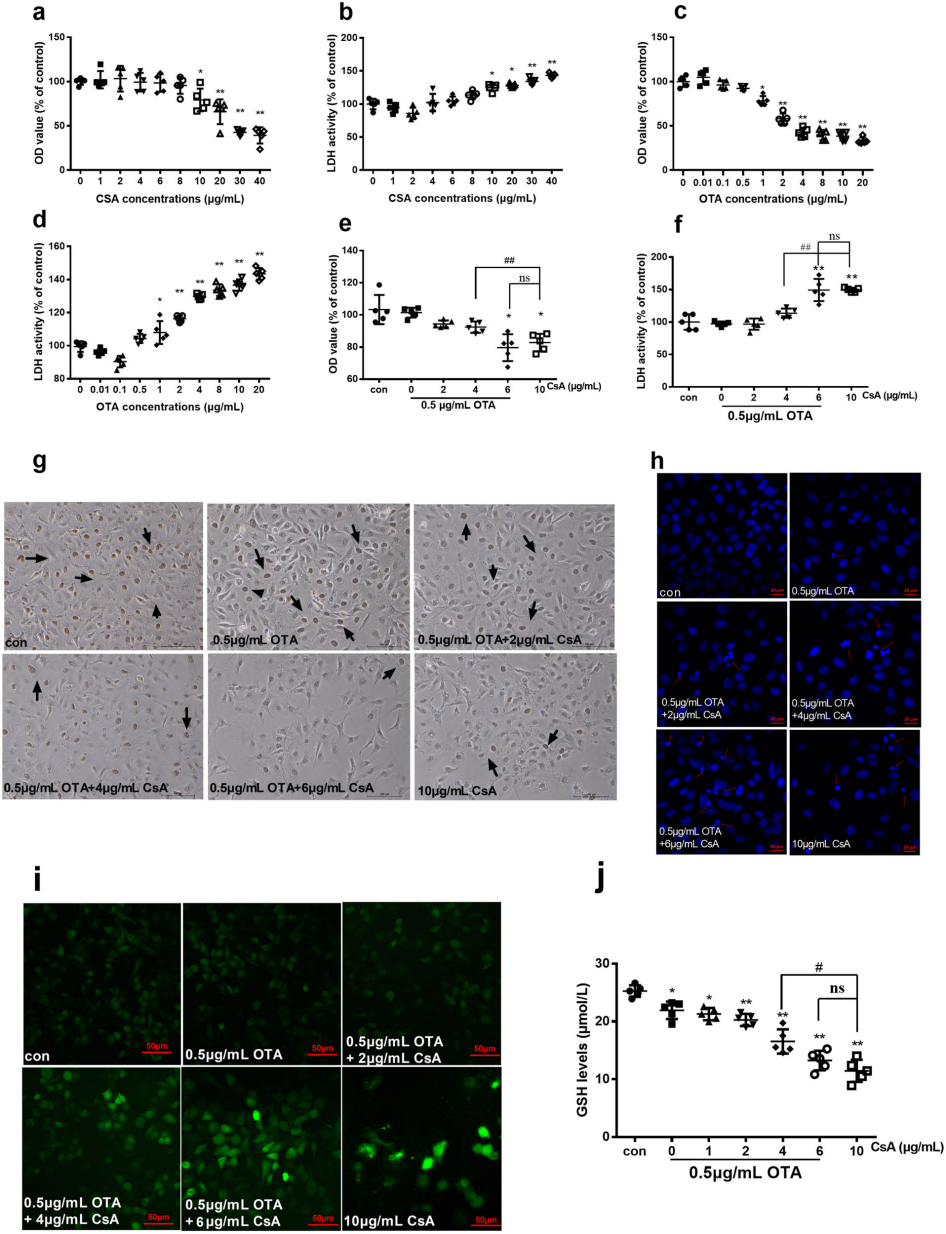
Nontoxic OTA decreased the concentration of CsA to induce cytotoxicity and oxidative stress in HK-2 cells. Cytotoxicity and oxidative stress were demonstrated by MTT **a**, **c**, **e**, LDH activity **b**, **d**, **f**, cell proliferation **g**, cell apoptosis **h**, ROS levels **i**, and production of GSH **j** after treatment of OTA or/and CsA for 48 h. All results were expressed as means ± SD (*n* = 5, *N* = 3). Significance compared with the control group, *P < 0.05; significance compared with the group of 10 μg/mL CsA, ^#^*P* < 0.05.

Cells were incubated with CsA (0, 1, 2, 4, 6, 8, 10, 20, 30, and 40 μg/mL) or OTA (0.01, 0.1, 0.5, 1, 2, 4, 8, 10, and 20 μg/mL) for 48 h. Results of MTT and LDH activities assay showed that cell viability was significantly decreased in a dose-dependent manner from the concentration of 10 μg/mL CsA or 1 μg/mL OTA (Fig. 3a–d). Hence, 0.5 μg/mL OTA without cytotoxicity was selected to combine with different concentrations of CsA (2, 4, and 6 μg/mL, which were <10 μg/mL). As shown in Fig. 3e, f, compared with 10 μg/mL CsA, combination of 0.5 μg/mL OTA and 6 μg/mL CsA had no significant difference. In Fig. 3g, h, cell proliferation capacity was decreased and apoptosis of cell nucleus was increased in a dose-dependent manner. Besides, intracellular ROS level was higher in combination of 0.5 μg/mL OTA and 6 μg/mL CsA than that in 10 μg/mL CsA, and the production of GSH was decreased in Fig. 3i, j. According to the results, it indicated that involvement of nontoxic OTA decreased the concentration of CsA to induce cytotoxicity and oxidative stress.

### Nontoxic concentration of OTA decreased the concentration of CsA to induce nephrotoxicity in vitro

**Fig. 4:**
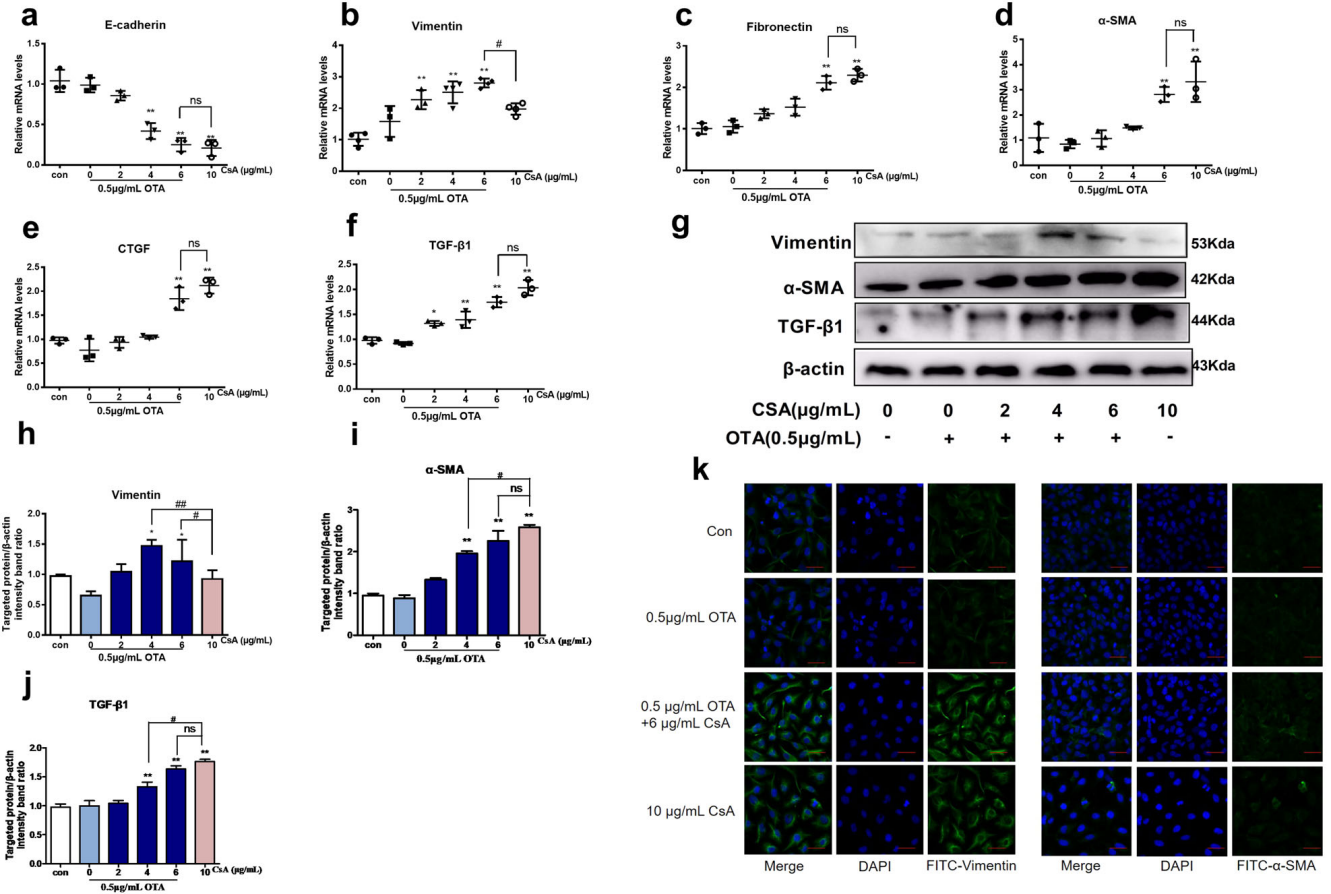
Nontoxic OTA decreased the concentration of CsA to induce nephrotoxicity in HK-2 cells. Cells were treated with OTA or/and CsA for 48 h. Nephrotoxicity related genes **a**–**f** and proteins **g**–**j** expressions were detected by western blotting and real-time PCR. And immunofluorescence staining of vimentin and α-SMA **k** was performed by laser confocal microscopy. All results were expressed as means ± SD (*N* = 3). Significance compared with the control group, *P < 0.05; significance compared with the group of 10 μg/mL CsA, ^#^*P* < 0.05.

In present chronic nephropathy model in mice, renal failure was observed that was characterized by renal fibrosis and tubular atrophy. To assess renal fibrosis in vitro, cells were treated with 0.5 μg/mL OTA combined with different concentrations of CsA (2, 4, and 6 μg/mL) or 10 μg/mL CsA alone. In Fig. 4, expression of E-cadherin was significantly reduced inversely, while expressions of fibronectin, α-SMA, Vimentin, connective tissue growth factor (CTGF), and TGF-β1 were significantly increased in the combination group of 0.5 μg/mL OTA and 6 μg/mL CsA. There was no significant difference between the combination group of 0.5 μg/mL OTA and 6 μg/mL CsA, and the group of 10 μg/mL CsA. In addition to the expression changes, localizations of vimentin and α-SMA were also observed in Fig. 4k. The staining areas of vimentin and α‐SMA were much more in the combination group and the group of 10 μg/mL CsA than that of control group and group of 0.5 μg/mL OTA.

### Nontoxic OTA and low concentrations of CsA in combination activated TLR4 and induced autophagy in vitro

**Fig. 5:**
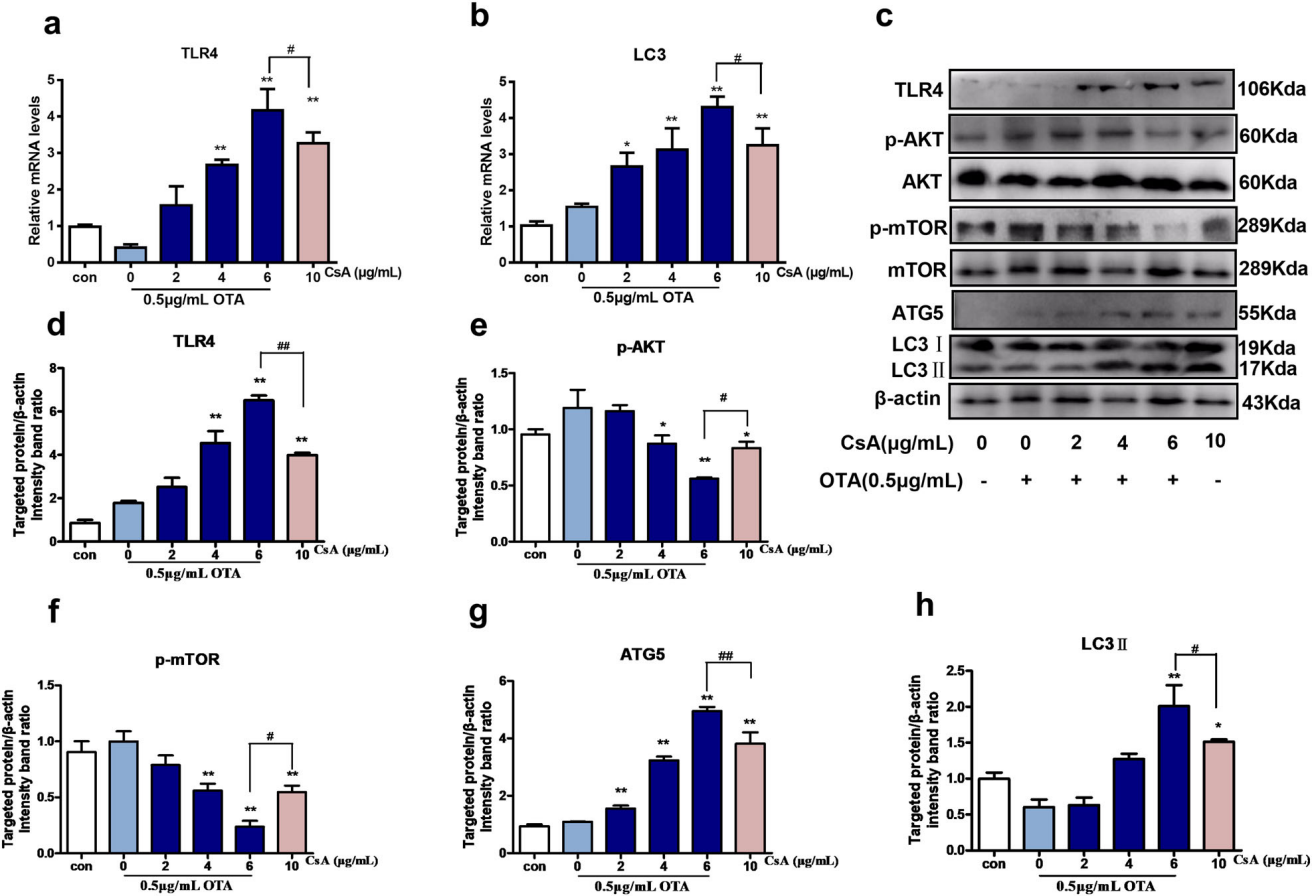
Low concentrations combination of OTA and CsA activated TLR4 and induced autophagy in HK-2 cells. Cells were treated with OTA or/and CsA for 48 h. Relative mRNA levels of TLR4 **a** and autophagy-related gene LC3 **b** were measured. TLR4 and autophagy-related proteins were tested **c**–**h**. All results were expressed as means ± SD (*n* = 3). Significance compared with the control group, *P < 0.05; significance compared with the group of 10 μg/mL CsA, ^*#*^*P* < 0.05.

TLRs are a main family that is recognized as a first line of innate defense against pathogen and endogenous signals of tissue injury. It has been verified that activation of TLR4 triggers an innate immune response and autophagy that is related to renal injury, and OTA can induce immune toxicity via ROS-relative TLR4 signaling pathway^21^. As shown in Fig. 5a–h, relative mRNA levels and protein expressions of TLR4, ATG5, and LC3II were markedly elevated as well as that AKT/mTOR signal pathway, which is an upstream pathway of autophagy was obviously inhibited in the combination group (0.5 μg/mL OTA + 6 μg/mL CsA) as compared with control group. According to the results, combination of 0.5 μg/mL OTA and 6 μg/mL CsA had no significant difference compared with 10 μg/mL CsA. The results indicated that the renal fibrosis induced by OTA and CsA could be related to TLR4 and autophagy. The combination group of 0.5 μg/mL OTA and 6 μg/mL CsA was selected to further investigate.

### Inhibition of autophagy alleviated the toxicities induced by combination of OTA and CsA in vitro

**Fig. 6:**
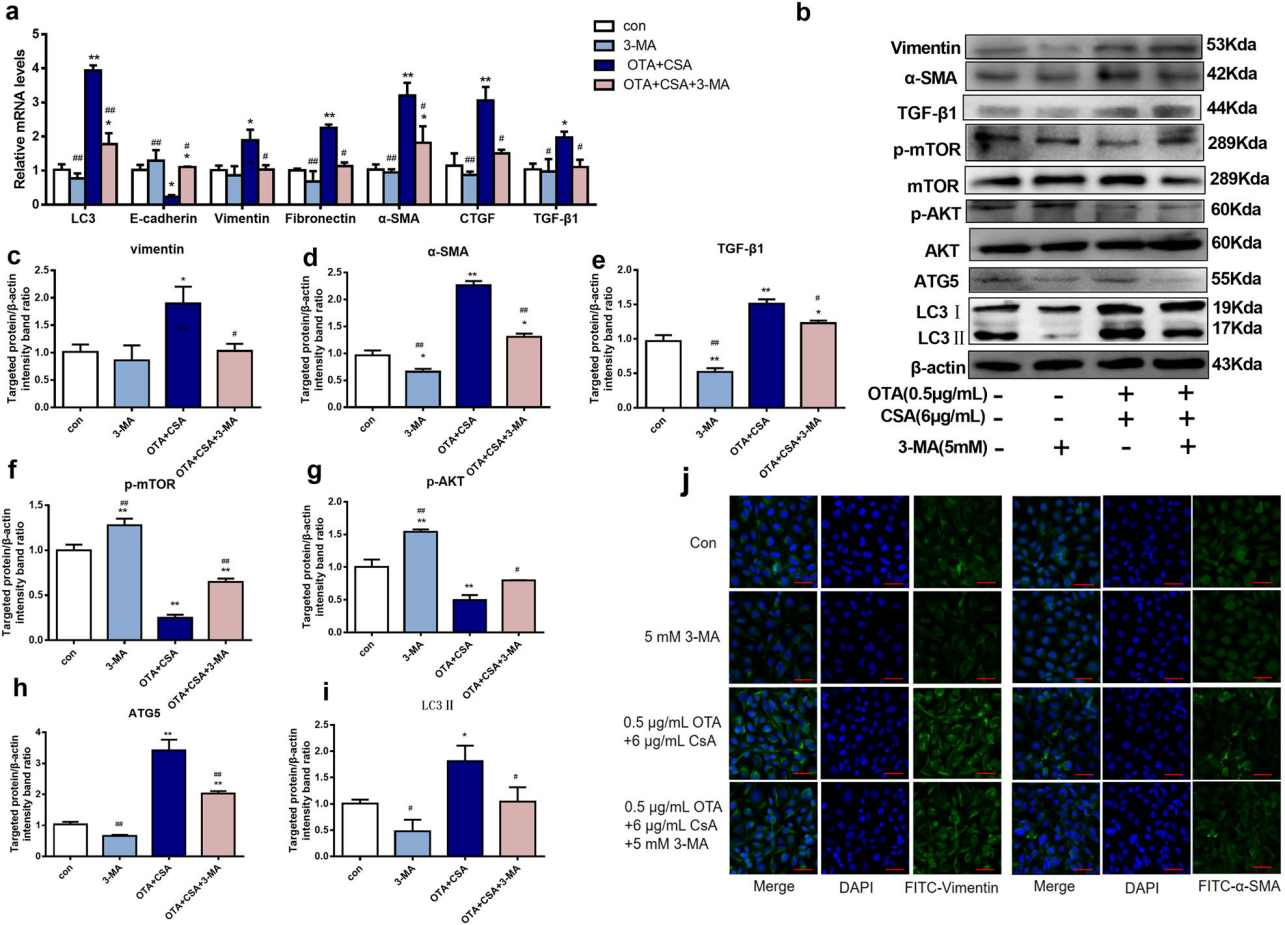
Inhibition of autophagy alleviated the toxicities induced by low concentrations combination of OTA and CsA in HK-2 cells. Cells were treated with OTA or/and CsA for 48 h. A total of 5 mM 3-MA as an inhibitor of autophagy was used at the same time. Relative mRNA levels of genes **a** were tested. Nephrotoxicity related proteins and autophagy-related proteins **b**–**i** were measured. Immunofluorescence staining of vimentin and α-SMA **j** was performed by laser confocal microscopy. All results were expressed as means ± SD (*n* = 3). Significance compared with the control group, *P < 0.05; significance of combination group compared with the combination group with 3-MA, ^*#*^*P* < 0.05.

To investigate whether autophagy is associated with renal injury induced by CsA and OTA, 3-methyladenine (3-MA, M9281) as an inhibitor of autophagy was used to investigate. The working concentration of 3-MA was 5 mM and it has no toxicity for cells (Supplementary Fig. 2a, b). In Fig. 6, it showed that treatment with 3-MA could significantly decreased the expressions of renal fibrosis-related proteins (Vimentin, α-SMA, and TGF-β1) and autophagy-related proteins (ATG5 and LC3II). The expressions of p-AKT, and p-mTOR were obviously elevated after treatment with 3-MA. In Fig. 6j, the staining intensity of Vimentin and α-SMA was much less after treatment with 3-MA. It indicated that contribution of OTA to CsA to induce fibrosis was accomplished through autophagy.

### Knockdown of TLR4 influenced autophagy and nephrotoxicity induced by combination of OTA and CsA in vitro

**Fig. 7:**
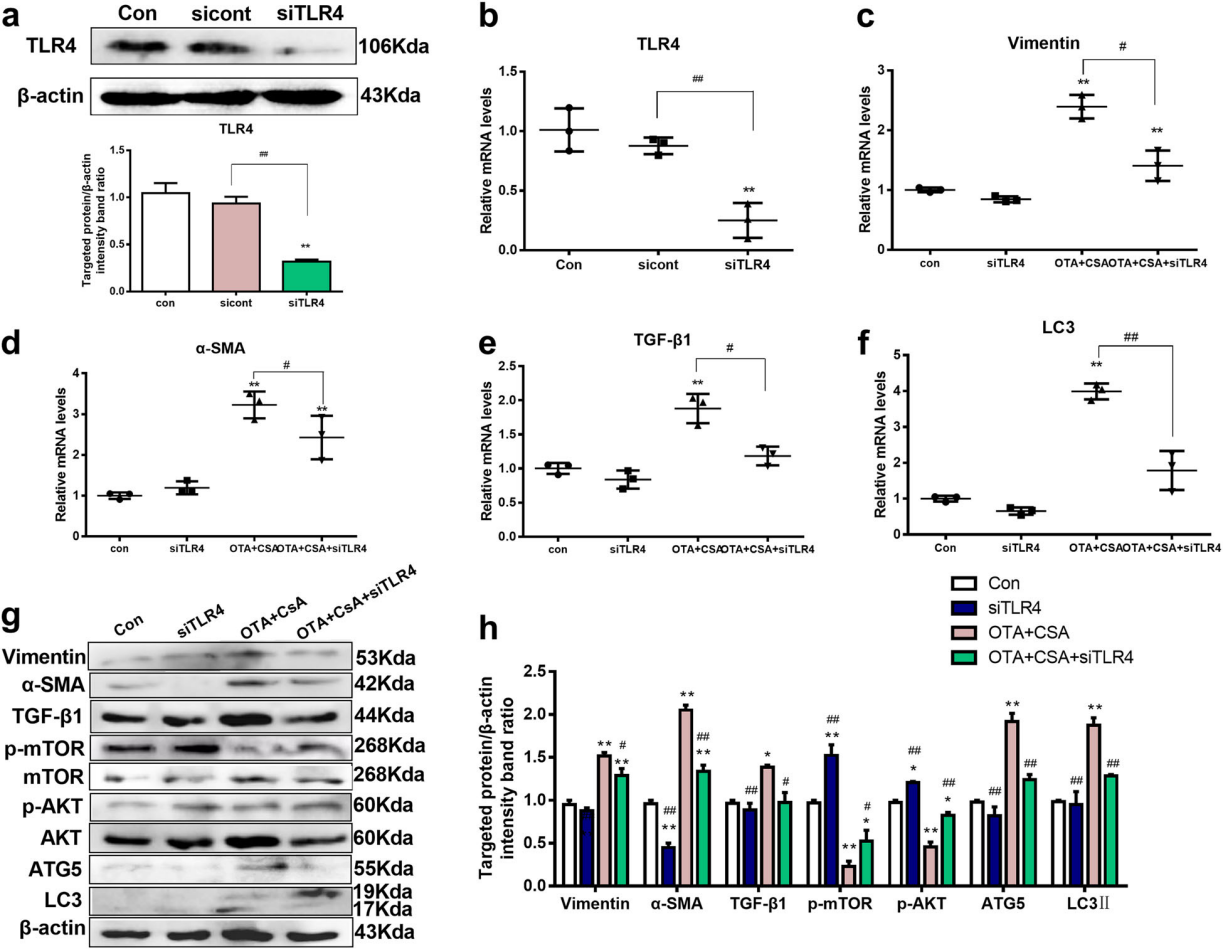
Knockdown of TLR4 influenced autophagy and the nephrotoxicity induced by low concentrations combination of OTA and CsA in HK-2 cells. Cells were cultured with siTLR4 for 6 h before treatment of OTA or/and CsA for 48 h. Transfection efficiency of TLR4 was tested by western blotting **a** and real-time PCR **b**. Changes of nephrotoxicity related genes **c**–**e** and autophagy-related genes **f** were detected. As well, the related proteins **g**, **h** were measured. All results were expressed as means ± SD (*n* = 3). Significance compared with the control group, *P < 0.05; significance of combination group compared with the combination group with siTLR4, ^*#*^*P* < 0.05.

In view of above results, it was further verified the relationship between TLR4 and autophagy. The knockdown of TLR4 was used to prove the influence on autophagy. TLR4-specific siRNA was used to remove its effects. The concentration of TLR4-specific siRNA had no effects on cells (Supplementary Fig. 2c, d). Meanwhile, the interfering efficiency of TLR4-specific siRNA was detected by real-time PCR and western blotting. As shown in Fig. 7a, b, the interfering efficiency was >70%. After knockdown of TLR4, expressions of p-mTOR and p-AKT were significantly elevated. Furthermore, expressions of fibrosis-related proteins (Vimentin, α-SMA, and TGF-β1) and autophagy-related proteins (ATG5 and LC3II) were reduced inversely in Fig. 7c–h. It indicated that combination of OTA and CsA-induced nephrotoxicity in human tubular cells (HK-2) via autophagy, which was mediated by TLR4.

### TLR4 deficiency attenuated the renal injury and autophagy in the chronic nephropathy model in vivo

**Fig. 8:**
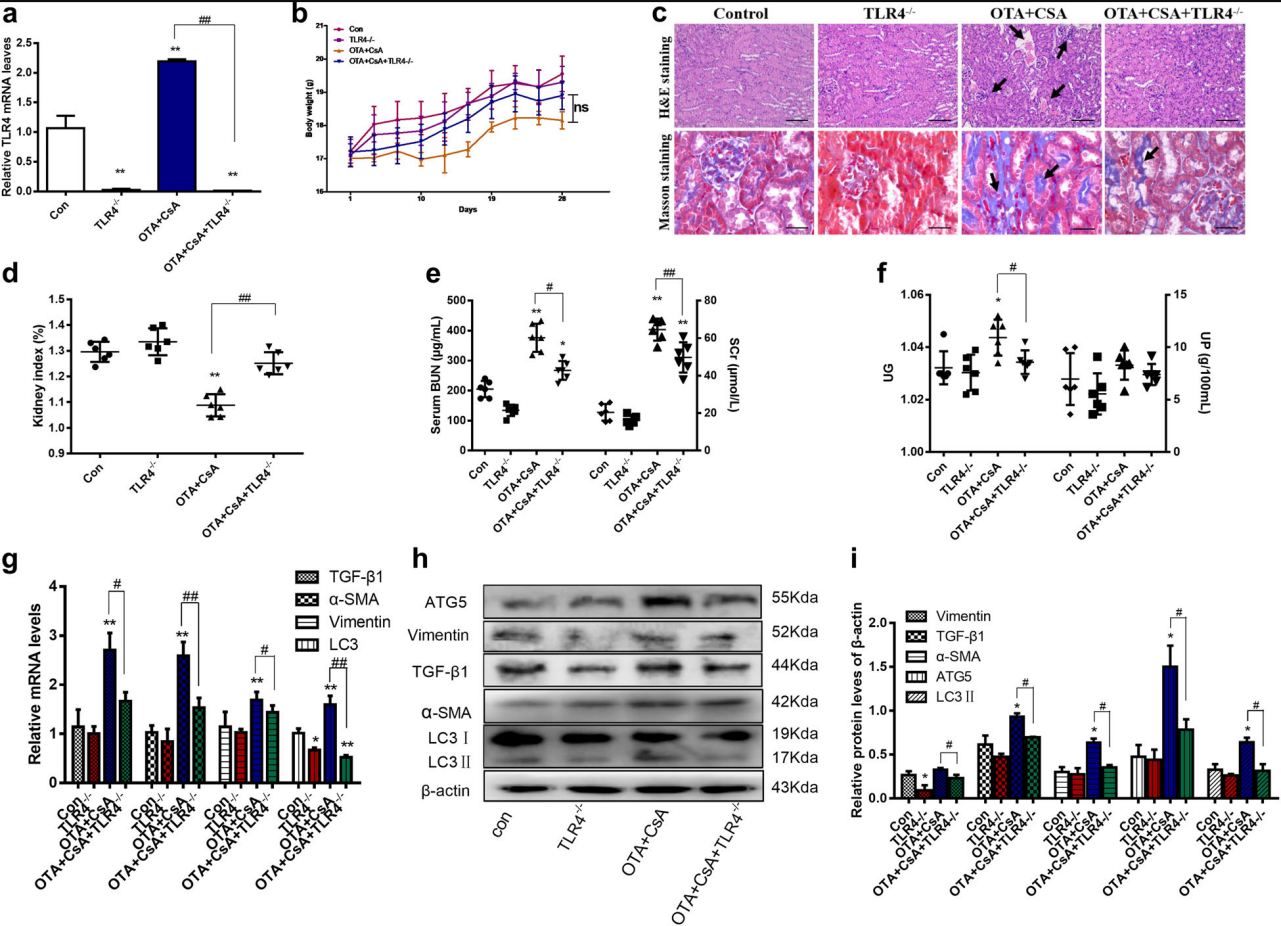
TLR4 deficiency attenuated the renal injury and autophagy in the chronic nephropathy model. The four groups of mice were control, TLR4^−/−^, OTA + CsA, and OTA + CsA + TLR4^−/−^. Mice were treated with OTA or/and CsA for 28 days. Relative mRNA levels of TLR4 **a** were measured. Body weight **b** was recorded and kidney index **d** were calculated, as the ratio of kidney weight and body weight. Pathological changes **c** of H&E (100×) and Masson (400×) staining were observed in kidneys. Serum BUN and Scr **e** were detected by corresponding kits. UG and UP **f** were measured by hand-held refractometer. Renal injury and autophagy related proteins **h**, **i** and relative mRNA levels **g** were tested by western blotting and real-time PCR. All results were expressed as means ± SD (*N* = 6, *n* = 3). Significance compared with the control group, *P < 0.05; significance of model group compared with the group of TLR4^−/−^ model group, ^*#*^*P* < 0.05.

TLR4^−/−^ mice were applied to further verify the mechanism in vivo. It was showed that TLR4 mRNA levels were obviously decreased in TLR4^−/−^ mice (Fig. 8a), and no significant difference was observed in body weight (Fig. 8b). Renal injury was decreased in H&E and Masson staining with reducing infiltration of inflammatory cells and expression of collagen I (Fig. 8c). Meanwhile, the decreases in kidney index (Fig. 8d), UG and UP (Fig. 8f) were reversed in TLR4^−/−^ mice. The increases of serum BUN, Scr (Fig. 8e), renal fibrosis-related genes and proteins (Vimentin, α-SMA, and TGF-β1), and autophagy-related genes and proteins, (ATG5 and LC3II) were inhibited in TLR4^−/−^ mice (Fig. 8g–i). It suggested that the renal injury and autophagy induced by OTA and CsA could be attenuated in TLR4 knockout mice.

## Discussion

CsA, which is widely used in transplant surgery and autoimmune diseases, causes nephrotoxicity by targeting renal tubular epithelial cells^26^. Because of its nephrotoxicity, CsA is applied to develop chronic nephropathy in rodent, rabbit, and pig model^5,6^. It was reported in previous investigations that application of 30 mg/kg CsA for 28 d could lead to chronic nephropathy in mice^27,28^. However, practice of CsA is restricted because of its side effects on heart, liver, and nervous system. The target organ of OTA is also kidney and several studies showed that OTA could cause renal injury in cells and animals^20,29^. And the dosage in our study is 0.25 mg/kg per day, which is nontoxic for mice. Therefore, we investigated that if nontoxic OTA could contribute to CsA to develop a chronic nephropathy model, reduce the dosage of CsA simultaneously to lessen the adverse effects, and explore the possible mechanism.

A number of studies of OTA provided that a decrease of body weight, and increases of creatinine and urea nitrogen levels in serum as well as renal fibrosis^30,31^. It was also reported that viability and junction structure of proximal tubular cells were disrupted as well as increasing productions of TGF-β1 and α-SMA, which were recognized as biomarkers of renal fibrosis^18,32^. TGF-β1 is a critical protein to promote renal fibrosis via activation of its downstream proteins^33,34^. Renal fibrosis usually leads to renal failure in the final stages of chronic kidney disease. Many factors, including α-SMA, E-cadherin, and TGF-β1 could affect extracellular matrix deposition and induce renal fibrosis. It was found that renal fibrosis is alleviated by enhancing expression of E-cadherin and depressing expressions of TGF-β1 and α-SMA^35^. It’s consistent with that combination of nontoxic OTA (0.25 mg/kg per day, qod) and low dosage of CsA (20 mg/kg per day) could induce renal fibrosis by upregulating expressions of Vimentin, TGF-β1, and α-SMA what is same as the conventional high dosage of chronic CsA model (30 mg/k per day), but the individual CsA (20 mg/kg per day) did not induce. These changes related to renal fibrosis were observed in the combination of OTA (0.5 μg/ml) and CsA (6 μg/ml), which is non-significant with individual CsA (10 μg/ml) as compared with control group in vitro.

On the other hand, long-time and massive intake of CsA has adverse effects. In some instances, it was reported that CsA had side effects on liver and cardiovascular, such as changes of bilirubin or hepatic aminotransferase levels^36,37^. We hypothesized that adverse effects could be alleviated through the contribution of nontoxic OTA. As expected, impairments of spleen, liver, and heart were observed in traditional chronic CsA (30 mg/kg per day) model, while it was mitigated in combined group that caused renal fibrosis at the same time. Besides, neurological symptoms were found in the group of 30 mg/kg per day CsA, where a mouse appeared to move in circles and died at the last day of experimental treatment, but there was no such sign in the other groups. The increased levels of AST, ALT, CK, and LDH in serum were decreased in combined group (0.25 mg/kg per day OTA + 20 mg/kg per day CsA). These changes in enzyme activities represent cardiac and hepatic functions. In addition, immunosuppressive action of CsA has side effects on chronic nephropathy model so we detected the status of immune organ. It was interesting that immunosuppressive status with 30 mg/kg per day CsA was mitigated by combined treatment of 0.25 mg/kg per day OTA + 20 mg/kg per day CsA. However, activation of TLR4 was found in renal. It may due to the immunosuppression of CsA mainly by suppressing humoral and cellular immunity, and the spleen is an immune organ, while the kidney is not.

We further investigated that the underlying mechanism of the contribution of OTA to CsA. In our previous study, low concentration of OTA could induce immune response through the ROS-relative TLR4/MyD88 signaling pathway and cause autophagy in vitro^25,38^. It proved that OTA could induce nephrotoxicity via autophagy in vivo and in vitro^19^. Several researches showed that TLR4 could mediate autophagy, and suppressed the apoptosis and autophagy by inhibition of the TLR4/MyD88 pathway in hippocampal neurons of epilepsy mice model^39,40^. The expressions of TLR4 and autophagy-related proteins were aggravated more in combined group than that in traditional chronic CsA model group. The effects could be reversed by autophagy inhibitor 3-MA or knockdown of TLR4 in vitro. TLR4^−/−^ mice were used to further verify in vivo, and we found that renal injury and autophagy induced by combination of OTA and CsA were obviously reversed in TLR4^−/−^ mice.

In conclusion, a chronic nephropathy model had been successfully developed by administration of nontoxic concentration of OTA and low dosage of CsA via TLR4-mediated autophagy. The side effects of the current model were significantly lesser than these of the previous model induced by onefold CsA. It is likely to be more beneficial for the development of specific treatment of chronic kidney disease.

## Supplementary information

Figure S1

Figure S2

Supplementary figures and legends

Video 1

Mechanistic figure
