# Supplementary figures and images for: Nontoxic concentration of ochratoxin A decreases the dosage of cyclosporine A to induce chronic nephropathy model via autophagy mediated by toll-like receptor 4
Source: Cell Death Dis. 2020 Feb 27;11(2):153. doi: 10.1038/s41419-020-2353-z (PMC7046648; doi:10.1038/s41419-020-2353-z)

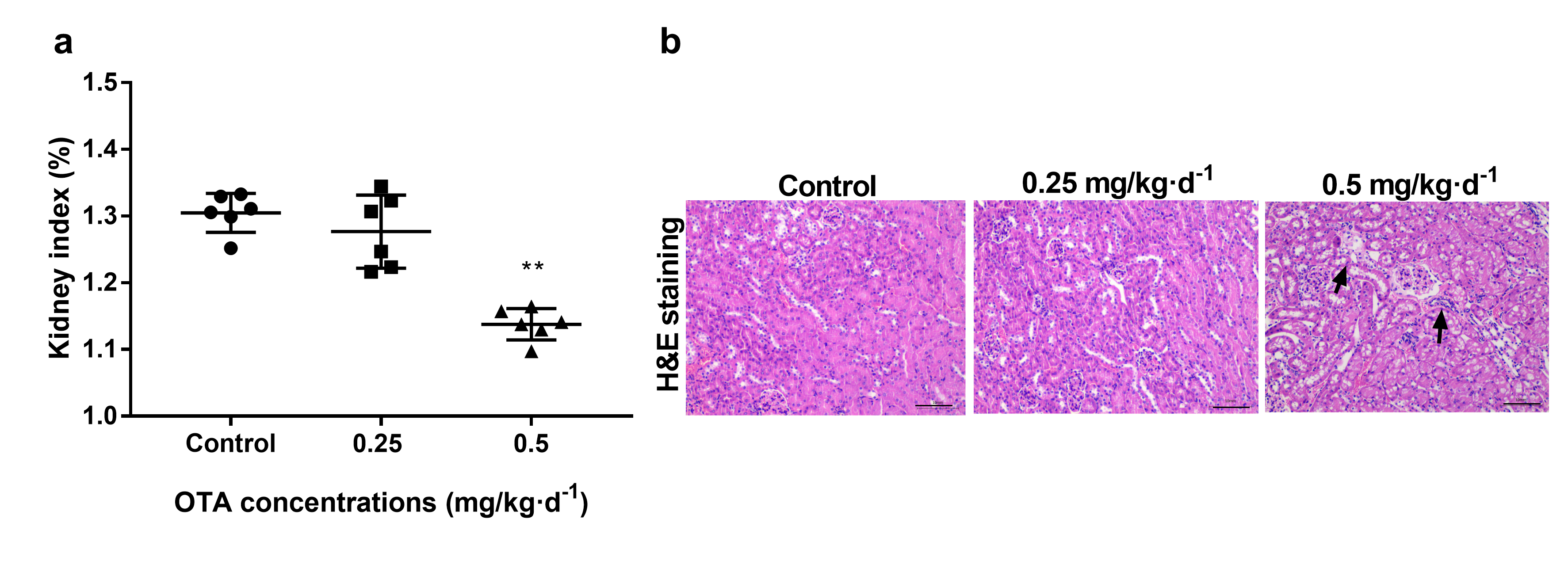

Supplement: Supplementary file 1 — Figure S1 [file 41419_2020_2353_MOESM1_ESM.tif]

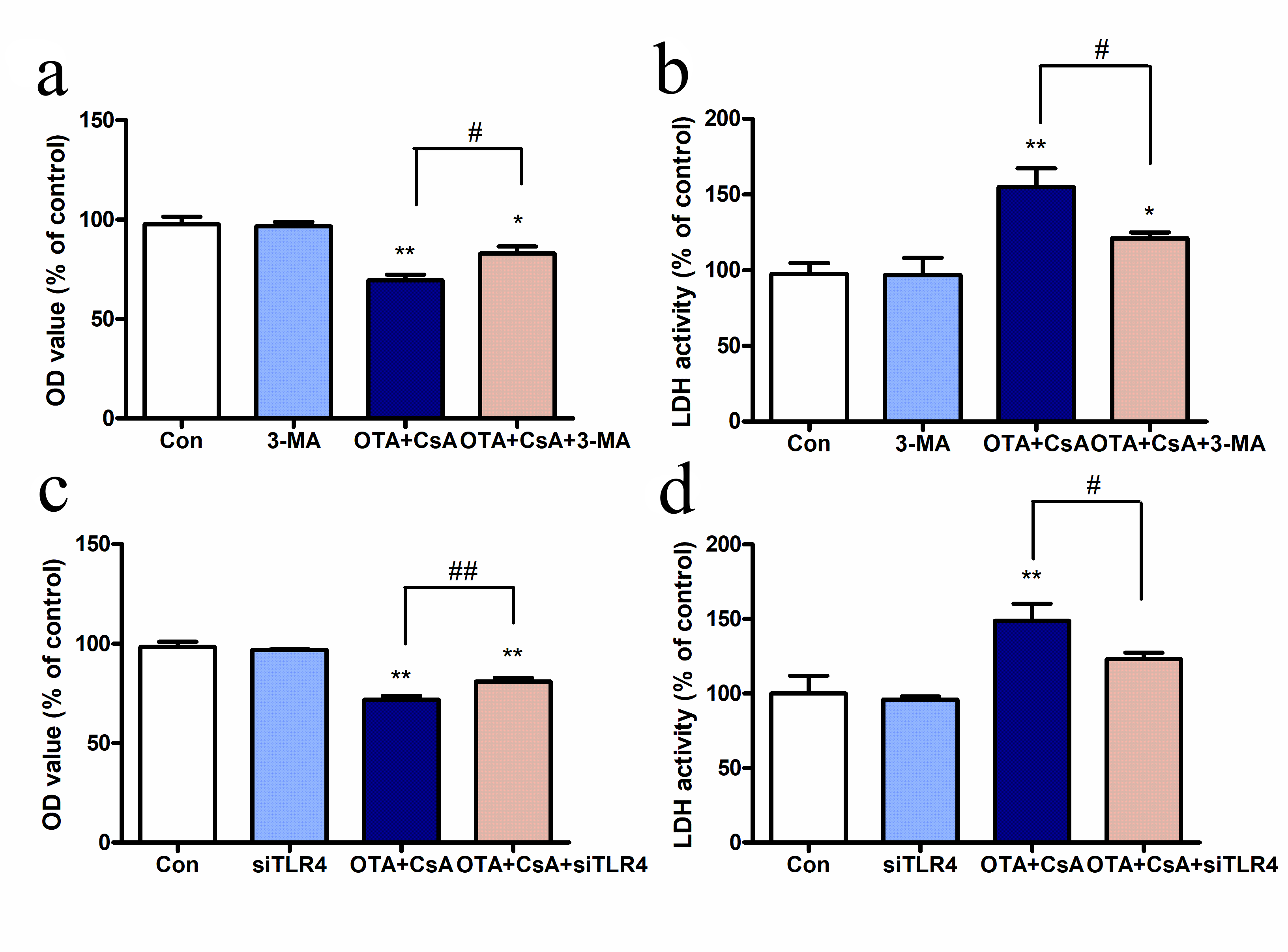

Supplement: Supplementary file 2 — Figure S2 [file 41419_2020_2353_MOESM2_ESM.tif]

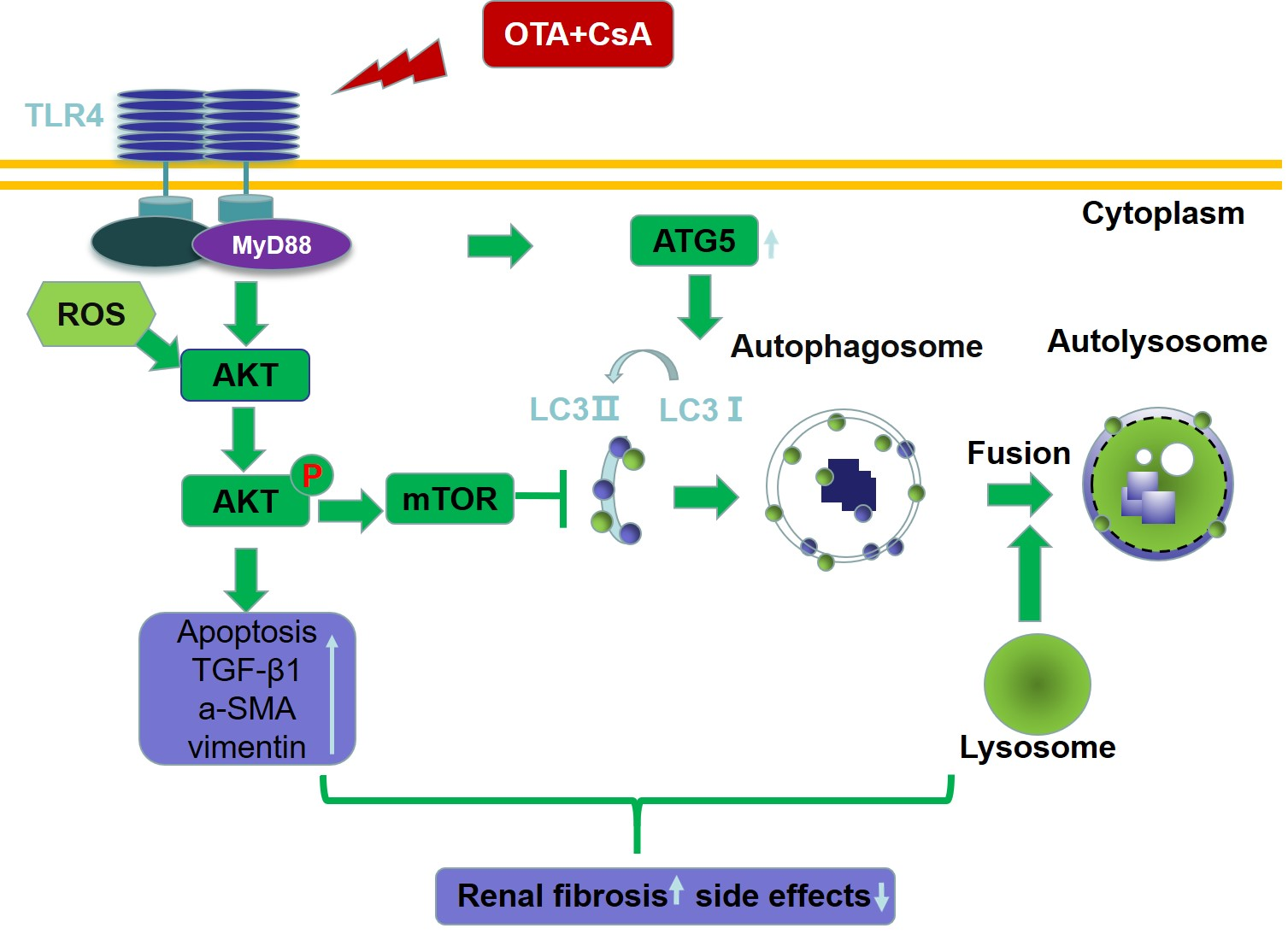

Supplement: Supplementary file 5 — Mechanistic figure [file 41419_2020_2353_MOESM5_ESM.tif]
