# Supplementary material for: Nontoxic concentration of ochratoxin A decreases the dosage of cyclosporine A to induce chronic nephropathy model via autophagy mediated by toll-like receptor 4
Source: Cell Death Dis. 2020 Feb 27;11(2):153. doi: 10.1038/s41419-020-2353-z (PMC7046648; doi:10.1038/s41419-020-2353-z)
Supplement: Supplementary file 3 — Supplementary figures and legends [file 41419_2020_2353_MOESM3_ESM.docx]

**
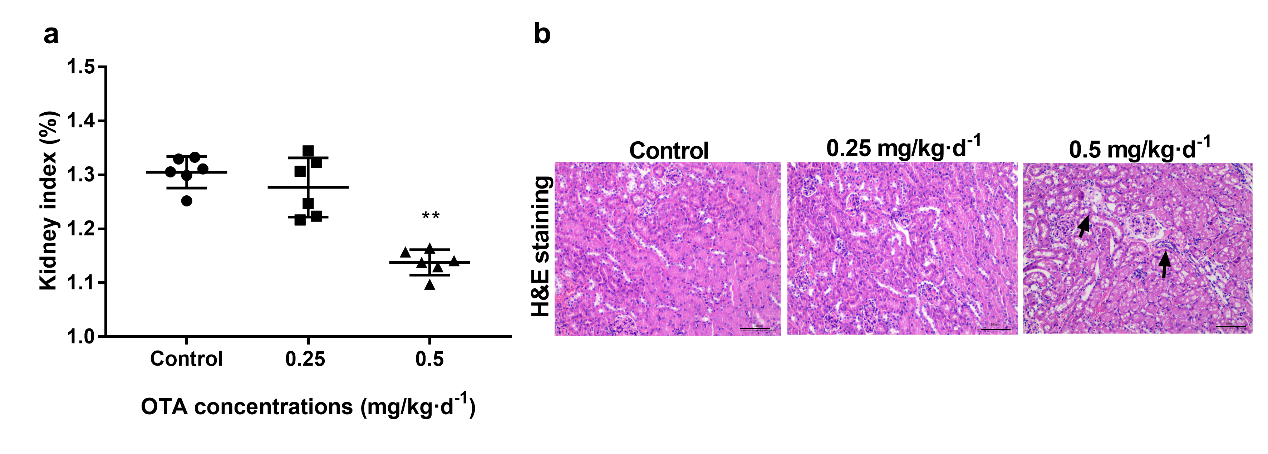
Fig S1** Effects of different dosages of OTA on renal injury. Mice were treated with or without OTA for 28 days. Kidney index **(a)** were calculated as the ratio of kidney weight and body weight. Pathological changes **(b)** were shown by H&E (100ⅹ) staining in kidneys. All results were expressed as means ± SD (n = 6). Significance compared with the control group, * *P < 0.05,* ** *P < 0.01*.

**
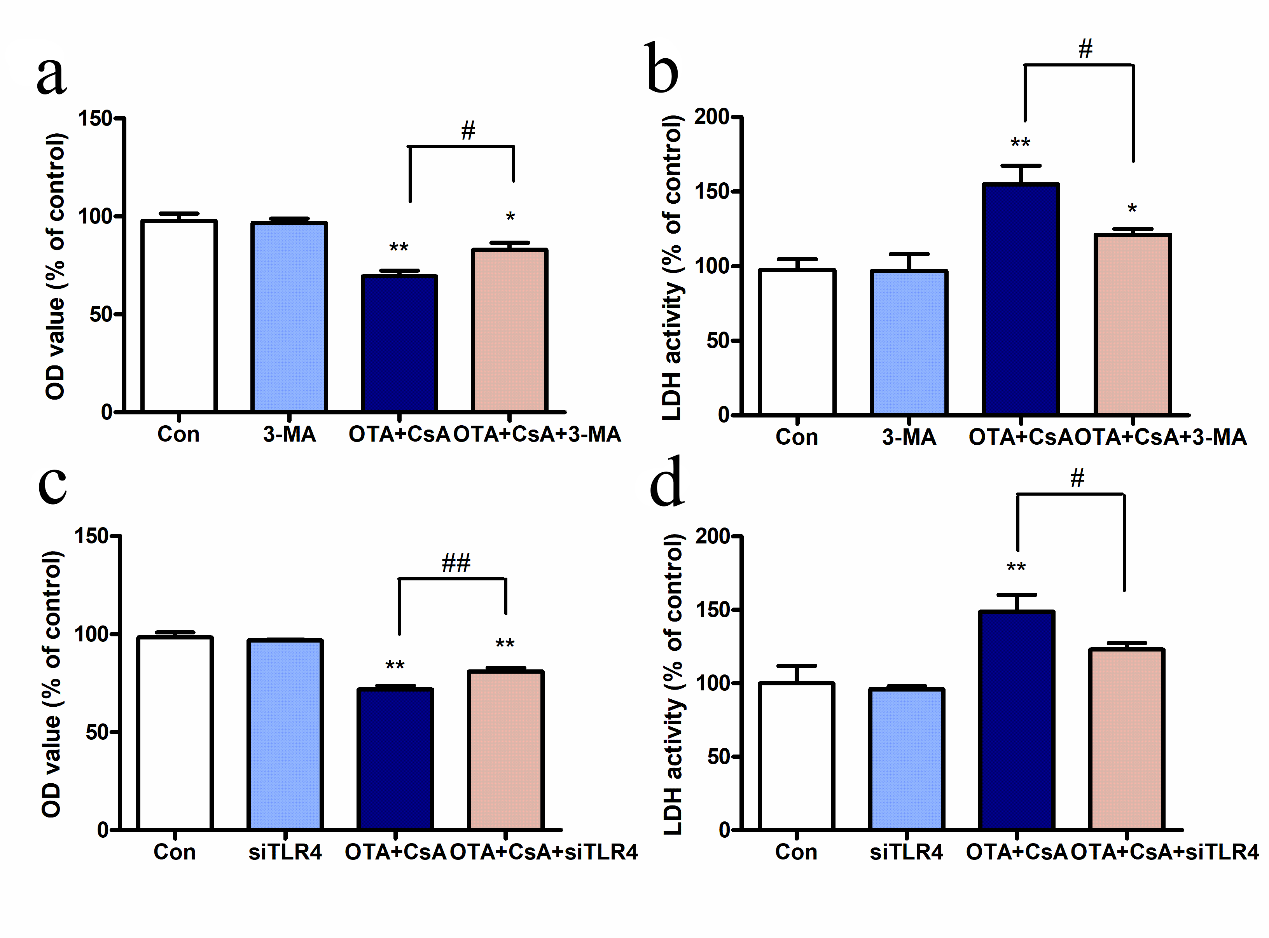
Fig S2** Effects of 3-MA and siTLR4 on cell viability. Cells were cultured after corresponding treatment. Cells were treated with OTA or/and CsA for 48h. MTT assay was used to detect cell survival situation **(a, c)**. And LDH activity **(b, d)** was measured by LDH kits. All results were expressed as means ± SD (N = 6). Significance compared with the control group, * *P < 0.05*; Significance of combination group compared with the combination group with 3-MA or siTLR4, *#* *P < 0.05.*
